# Platelet-Rich Plasma Releasate versus Corticosteroid for the Treatment of Discogenic Low Back Pain: A Double-Blind Randomized Controlled Trial

**DOI:** 10.3390/jcm11020304

**Published:** 2022-01-07

**Authors:** Koji Akeda, Kohshi Ohishi, Norihiko Takegami, Takao Sudo, Junichi Yamada, Tatsuhiko Fujiwara, Rui Niimi, Takeshi Matsumoto, Yuki Nishimura, Toru Ogura, Satoshi Tamaru, Akihiro Sudo

**Affiliations:** 1 Department of Orthopaedic Surgery, Mie University Graduate School of Medicine, Tsu 514-8507, Japan; n-takegami@clin.medic.mie-u.ac.jp (N.T.); takao.vb8@gmail.com (T.S.); yamada-j@med.mie-u.ac.jp (J.Y.); tatsuhiko-f@clin.medic.mie-u.ac.jp (T.F.); a-sudou@clin.medic.mie-u.ac.jp (A.S.); 2 Department of Transfusion Medicine and Cell Therapy, Mie University Hospital, Tsu 514-8507, Japan; koishi@clin.medic.mie-u.ac.jp (K.O.); matsutak@clin.medic.mie-u.ac.jp (T.M.); 3 Niimi Orthopedics Clinic, Kuwana 511-0934, Japan; furikakefuri@hotmail.co.jp; 4 Clinical Research Support Center, Mie University Hospital, Tsu 514-8507, Japan; ynishimura@clin.medic.mie-u.ac.jp (Y.N.); t-ogura@clin.medic.mie-u.ac.jp (T.O.); tamaru3@clin.medic.mie-u.ac.jp (S.T.)

**Keywords:** intervertebral disc degeneration, platelet-rich plasma, corticosteroid, low back pain

## Abstract

Clinical application of platelet-rich plasma is gaining popularity in treating low back pain (LBP). This study investigated the efficacy and safety of platelet-rich plasma releasate (PRPr) injection into degenerated discs of patients with discogenic LBP. A randomized, double-blind, active-controlled clinical trial was conducted. Sixteen patients with discogenic LBP received an intradiscal injection of either autologous PRPr or corticosteroid (CS). Patients in both groups who wished to have PRPr treatment received an optional injection of PRPr eight weeks later. The primary outcome was change in VAS from baseline at eight weeks. Secondary outcomes were pain, disability, quality of life (QOL), image analyses of disc degeneration, and safety for up to 60 weeks. The VAS change at eight weeks did not significantly differ between the two groups. Fifteen patients received the optional injection. Compared to the CS group, the PRPr group had a significantly improved disability score at 26 weeks and walking ability scores at four and eight weeks. Radiographic disc height and MRI grading score were unchanged from baseline. PRPr caused no clinically important adverse events. PRPr injection showed clinically significant improvements in LBP intensity equal to that of CS. PRPr treatment relieved pain, and improved disability and QOL during 60 weeks of observation.

## 1. Introduction

The Global Burden of Diseases, Injuries, and Risk Factors Study 2017 (GBD 2017) [1] conducted in 195 countries for 354 medical conditions reported that low back pain (LBP) was the leading cause of worldwide productivity loss and disability, with enormous socioeconomic and health impacts [2]. Among the anatomical elements comprising the lumbar spine, intervertebral disc (IVD) degeneration is one of the major causes of LBP [3]; this is termed ‘discogenic LBP’.

IVD degeneration is accompanied by cellular and extracellular matrix changes in intradiscal microenvironments that eventually lead to structural breakdown and impaired IVD function [4]. Aberrant expression of proinflammatory cytokines found in degenerated human IVDs induces progressive degradation of major extracellular matrix components, including proteoglycan and type II collagen, by stimulating matrix-degrading enzymes [4]. Notably, proinflammatory stimuli also enhance the expression of nociceptive molecules within degenerated IVDs affecting sensory endings in the outer layer of the annulus fibrosus [3]. Therefore, matrix degradation with nociceptive stimuli in the inflammatory microenvironment is responsible for discogenic pain.

Bioactive factors, including growth factors [5,6,7] and related molecules [8], have been extensively evaluated in vitro and vivo for clinical utility in IVD repair (see reviews in [9,10]). Platelet-rich plasma (PRP) is an autologous blood concentrate containing a majority of bioactive molecules [11]. PRP promotes tissue repair and cellular growth by anabolic effects of several growth factors released from activated platelets [11,12]. It also exerts anti-inflammatory properties that modulate tissue repair processes and are related to analgesic effects [13,14]. Recently, PRP has been used with increasing frequency for the treatment of musculoskeletal pathologies, including muscle and tendon injuries and osteoarthritis [11]. A recent meta-analysis of randomized controlled clinical trials (RCTs) reported that intra-articular injection of PRP is effective for pain relief and functional improvement in knee osteoarthritis [15].

PRP has been shown to activate metabolism of IVD cells in vitro, and induce reparative effects on IVD degeneration in animal studies [11], suggesting that intradiscal injection of PRP is a promising therapy to repair IVD degeneration.

Clinical studies of intradiscal PRP injection treatment for patients with LBP have been reported [11] and a meta-analysis of 12 clinical studies showed significant improvements in LBP [16]. However, there has been only one RCT by Tuakli-Wosornu et al. [17]. In 2017, we conducted a preliminary clinical trial to evaluate the effect of the releasate isolated from PRP (PRPr) in 14 patients with discogenic LBP and reported its safety and preliminary analgesic effect [18]; however, RCTs are needed to determine the clinical efficacy of PRPr. The purpose of this study was to evaluate the efficacy and safety of intradiscal injection of PRPr in comparison with corticosteroid injection in patients with discogenic LBP.

## 2. Materials and Methods

### 2.1. Study Design

**Figure 1:**
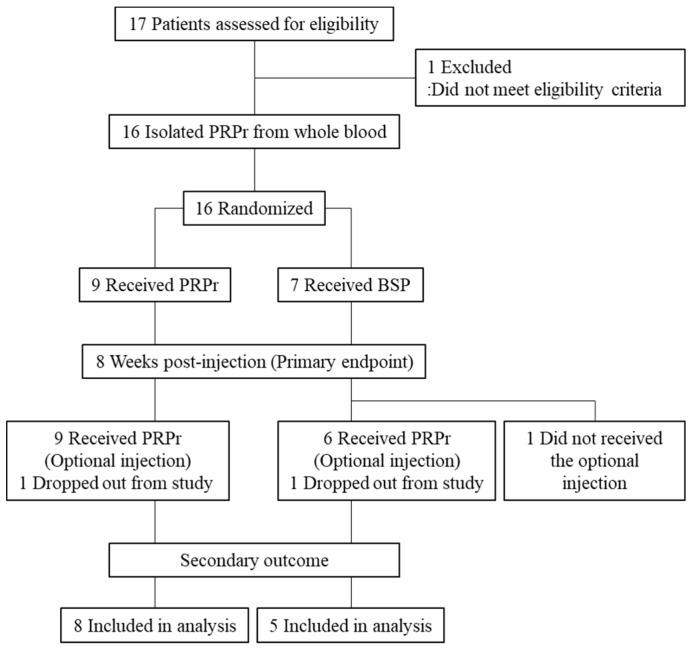
Flow of patients.

This study was a randomized, double-blind, active-controlled clinical trial conducted between February 2018 and September 2020 in a single institution. Patients were recruited via a rigorous selection process and received an intradiscal injection of either PRPr or the corticosteroid (CS). Patients from both the PRPr and CS groups who still experienced pain received PRPr as an optional treatment eight weeks post-injection (Figure 1). The efficacy and safety of PRPr were evaluated for up to 52 weeks after initial injection or optional injection. The institutional review board (IRB) approved this randomized controlled trial. All patients provided written informed consent.

### 2.2. Participants

Patients aged >18 years who had LBP for more than three months with one or more lumbar discs (L3/L4 to L5/S1) with evidence of degeneration, as indicated by magnetic resonance imaging (MRI), and at least one symptomatic disc, confirmed using standardized provocative discography, were considered for inclusion. The inclusion and exclusion criteria are shown in Table 1.

### 2.3. Randomization

Randomization of study patients in either the PRPr or the CS group on a 1:1 bias was performed in the institutional clinical research center. Stratification factors were not included in the randomization procedure. Both the outcome assessor and participants were blinded to group assignments.

### 2.4. PRP Releasate (PRPr) Preparation and Fluoroscopy-Guided Injection

PRPr isolation was performed as previously reported [18]. In short, autologous PRP was prepared from whole blood (400 mL) using the buffy coat method [22]. The supernatant isolated from activated PRP (PRPr) (approximately 15 mL) was stored at −20 °C until use.

Intravenous antibiotics (Cefazolin Sodium: 1 g) were administered within 60 min before the injection procedure. The injection site was treated with a local anesthetic (0.5% lidocaine). Under fluoroscopy, a 22 gauge, 150-mm spinal needle was inserted into the center of the targeted disc. PRPr (2 mL) or the CS (betamethasone sodium phosphate, Sionogi & Co., LTD, Osaka, Japan) (2 mg in 2.0 mL of saline) was injected through a syringe filter sterilization.

### 2.5. Outcome Measures

The intensity of LBP was measured by the visual analogue scale (VAS), which is reliable, valid, and sensitive to change in the evaluation of pain intensity [23,24,25]. For evaluation of patients’ LBP-related quality of life (QOL) and/or disability, all the patients completed the following patient-reported outcome measures, including Oswestry Disability Index (ODI) [26], Roland-Morris Disability Questionnaire (RDQ) [27,28], and Japanese Orthopaedic Association Back Pain Evaluation Questionnaire (JOABPEQ) [29,30,31]. Higher scores of ODI and RDQ indicate worse conditions, and those of JOABPEQ indicate better conditions.

The primary outcome was defined as the change from baseline in LBP evaluated using the VAS (0–100 mm) for PRPr versus (vs.) CS at week 8 post-injection.

Secondary outcomes included (1) the change (time points − baseline) and % change ([time points − baseline]/baseline × 100) in VAS, ODI, RDQ, and JOABPEQ at 4, 8, 12, 26, and 52 weeks after the injection from those at baseline; (2) change in radiographic disc height index (DHI) [21] at 4, 8, 12, 26, and 52 weeks after the injection from DHI at baseline; (3) change in the Pfirrmann [19] and modified Pfirrmann [32] grading system at 26 and 52 weeks after the injection from baseline; and (4) a successful ratio of the treatment at 8, 12, 26, and 52 weeks after the injection. Treatment success was defined as patients who met all of the following requirements: (1) Improvement of VAS by more than 30% from baseline (% change: less than −30%); (2) more than 30% improvement in ODI from baseline (% change: less than −30%); (3) no additional treatment; and (4) no serious adverse events (AEs) following PRPr administration. When the patients received the optional injection, the secondary outcomes were evaluated at 12, 16, 20, 34, and 60 weeks after the initial injection (4, 8, 12, 26, and 52 weeks after the optional injection).

The safety endpoints were AEs, including serious AEs, laboratory parameters, vital signs, and neurological symptoms.

### 2.6. Statistical Analysis

Based on the results of the feasibility study [18], a power analysis revealed that a sample size of nine patients per treatment group was necessary to detect a substantial difference between the PRPr and CS groups of approximately 17 mm in the VAS at week 8 with 80% power and a two-sided significance of 0.05.

All efficacy and safety analyses were performed on the full analysis set and safety analysis set. The change or % change in VAS, ODI, RDQ, DHI, and JOABPEQ were summarized by means and standard deviations (SDs) for each group were assessed by two-way repeated-measures analysis of variance (ANOVA). The differences in each time point analysis between groups were assessed by the unpaired t-test or Mann–Whitney U test. All statistical analyses were performed using SAS version 9.4 (SAS Institute Inc., Cary, NC, USA). The accepted level of significance was set at *p* < 0.05.

## 3. Results

### 3.1. Patient Characteristics

Sixteen patients (mean age: 32.2 ± 8.3 years old, 11 men, 5 women) were included in the study. The PRPr group included nine patients (mean age: 35.1 ± 8.7 years old, 6 men, 3 women). The CS group included seven patients (mean age: 27.9 ± 5.2 years old, 5 men, 2 women). A total of 21 discs (L3/L4: 3 discs, L4/L5: 12 discs, L5/S1: 6 discs) were targeted for treatment. Patient characteristics are summarized in Table 2. No significant differences were found in any baseline characteristics. The patient registration was canceled according to the advice from IRB based on the results of the interim analysis on the primary outcome. Hence, 16 patients enrolled were followed up over the observation period. One patient in the PRPr group and one in the CS group dropped out after 16 weeks because the patients opted for long-term use of analgesic drugs due to persistent low back pain.

Sixteen patients received either the releasate isolated from activated platelet-rich plasma (PRPr) or corticosteroid (CS). Patient-reported outcomes, including the visual analogue scale (VAS), Oswestry Disability Index (ODI) [26], Roland-Morris Disability Questionnaire (RDQ) [27], and Japanese Orthopaedic Association (JOA) Back Pain Evaluation Questionnaire (JOABPEQ) [30], were evaluated. Data were expressed as mean ± standard deviation (S.D.). The Pfirrmann [19] and modified Pfirrmann [32] grading system were used to assess the MRI grade of disc degeneration.

### 3.2. Quality Assessment of Platelet-Rich Plasma (PRP)

The mean platelet count of PRP was approximately 4.3 times greater than that of whole blood (whole blood: [257.0 ± 41.3] × 103 platelets/µL; PRP: [1095.2 ± 362.9] × 103 platelets/µL). The mean white blood cell (WBC) count of PRP was approximately 1/58 of whole blood (whole blood, [6.46 ± 1.52] × 103 cells/µL; PRP, [0.11 ± 0.14] × 103 cells/µL). The isolated PRP used in this study was classified as ‘pure PRP’ [13]. There were no significant differences in the platelet and WBC counts in whole blood and PRP between the two groups.

### 3.3. Primary Outcome of Efficacy

**Figure 2:**
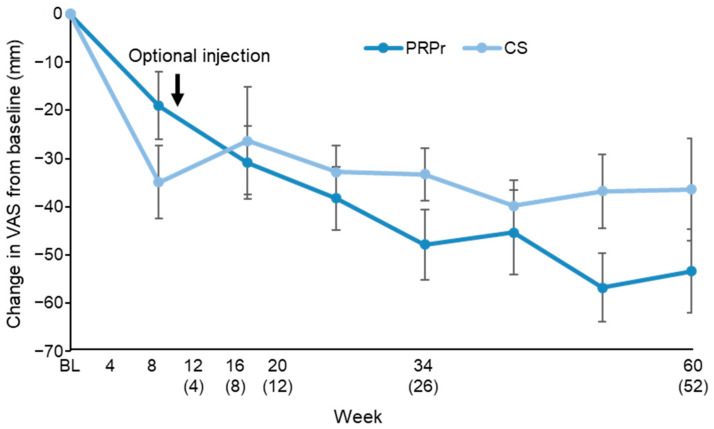
Change in the visual analogue scale (VAS). VAS was evaluated for 60 weeks after the injection of the releasate isolated from activated platelet-rich plasma (PRPr) or corticosteroid (CS). The number in parentheses indicates weeks after the optional injection. Data were expressed as means ± standard error of the means (SEMs).

The VAS scores of both the PRPr and CS groups decreased significantly over the observation period (*p* < 0.01), but those changes from baseline across all the observations did not differ significantly between the groups (*p* = 0.76, repeated measures ANOVA) (Figure 2).

The primary outcome showed that the mean change in the VAS score from baseline to week 8 was −30.9 mm in the PRP group and −26.3 in the CS group; there were no significant differences between the two groups (*p* = 0.61) (Figure 2).

### 3.4. Secondary Outcomes of Efficacy

#### 3.4.1. Outcomes of the Optional Injection (*n* = 15)

**Figure 3:**
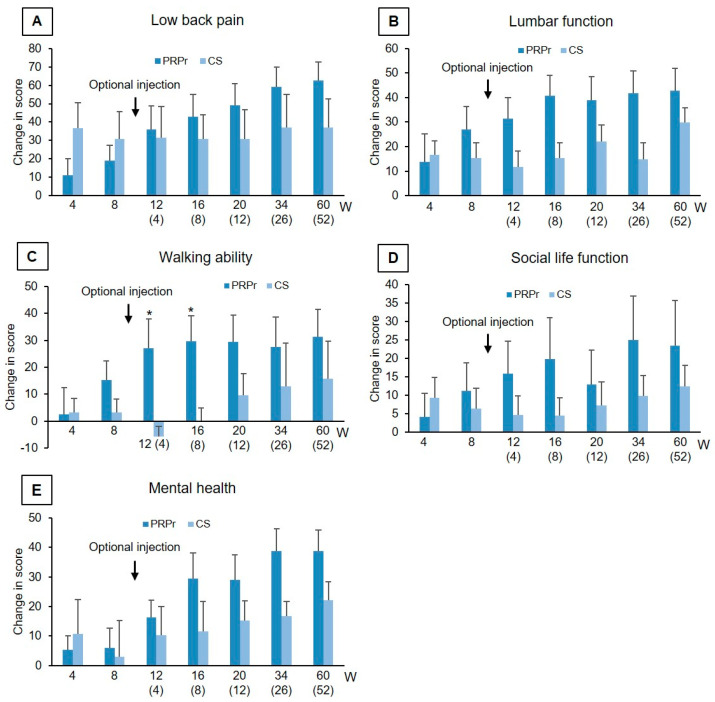
The Japanese Orthopaedic Association back pain evaluation questionnaire (JOABPEQ). JOABPEQ [30] is composed of 25 items across five domains: (**A**) Low back pain, (**B**) lumbar function, (**C**) walking ability, (**D**) social life function, and (**E**) mental health. All five domains of JOABPEQ were evaluated for 60 weeks after injection of the platelet-rich plasma releasate (PRPr) or corticosteroid (CS). The number in parentheses indicates weeks after the optional injection. Data were expressed as means ± standard error of the means (SEMs). * *p* < 0.05 vs. CS.

LBP, disability, and QOL: There were no significant differences in both the mean change and/or % change from baseline in VAS, ODI, RDQ (Table 3), and all five categories of JOABPEQ (Figure 3) at weeks 4 and 8 after the initial injection. In addition, no significant differences on the changes in VAS, ODI, RDQ, and all five items of JOABPEQ from baseline were also found between the subjects with one disc (*n* = 11) and two discs (*n* = 5) injection at week 8 after the initial injection.

After the optional injection of PRPr, the % change in RDQ in the PRPr group was significantly decreased compared to that in the CS group at week 26 post-optional injection (PRPr: −88.0 ± 23.5%, CS: −42.1 ± 45.1%, *p* < 0.05, Table 3). The mean change in the ‘walking ability’ of JOABPEQ in the PRPr group was significantly higher than the CS group at weeks 4 and 8 after the optional injection (*p* < 0.05, respectively, Figure 3C). Concerning other items, no significant differences were identified between the two groups throughout the observation period (Table 3).

Success rate: At week 8, the success rate of the PRPr group was 55.6%, while that of the CS group was 28.6% (*p* = 0.36). At week 60, the success rate was 87.5% in the PRPr group and 40% in the CS group; however, the difference was not statistically significant (*p* = 0.22).

**Figure 4:**
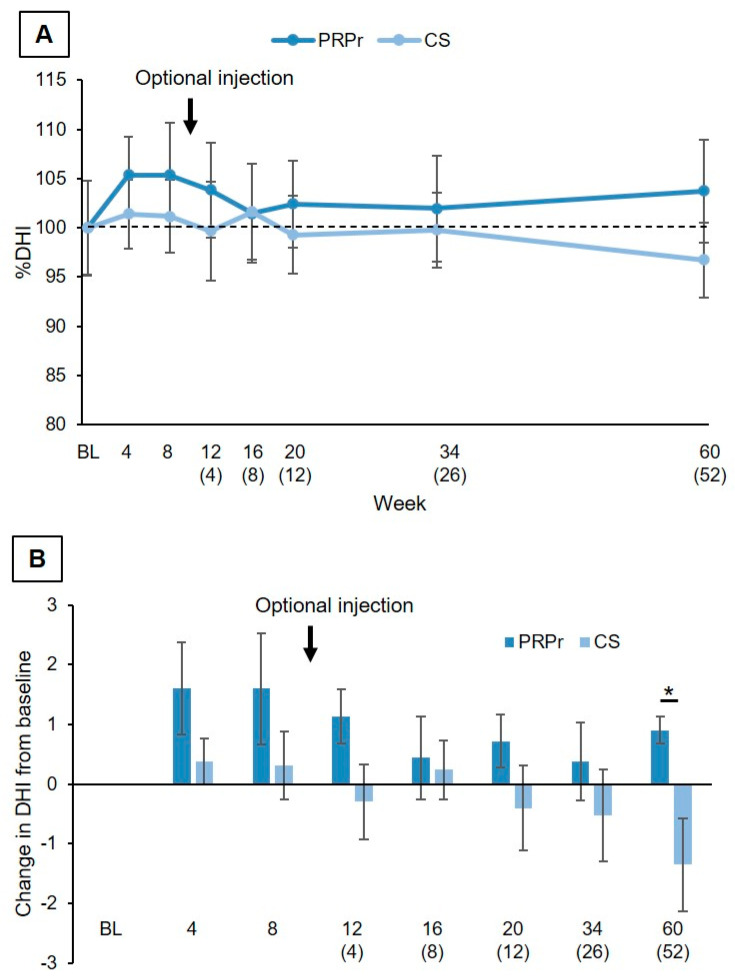
Change in disc height. (**A**) Percent disc height index (%DHI) was measured for 60 weeks after injection of the platelet-rich plasma releasate (PRPr) or corticosteroid (CS). The number in parentheses indicates weeks after the optional injection. Data were expressed as means ± standard error of the means (SEMs). (**B**) Change in disc height index (DHI) from baseline. * *p* < 0.05.

Change in DHI: % DHI (DHI at each time point/DHI at baseline × 100) of both the PRPr and CS groups did not show significant time-dependent changes over the observation period (*p* = 0.20) and did not differ significantly between the two groups (*p* = 0.74) (Figure 4A). The mean change in DHI of the PRPr group tended to be higher than that of the CS group throughout, and statistical significance was identified at week 60 (*p* < 0.05, Figure 4B).

MRI analysis: No patients showed changes in either the Pfirrmann [19] nor the modified Pfirrmann [32] grading scores at weeks 26 and 52 after the optional injection compared to scores at baseline in both groups.

#### 3.4.2. Outcomes of Single Injection (*n* = 1)

One patient in the CS group did not receive an optional injection. VAS, ODI, and RDQ decreased at week 8, and these were sustained over the observation period (changes from baseline: VAS: 8 W: −60 mm, 52 W: −56 mm; ODI (%): 8 W: −21.8, 52 W: −19.6; RDQ: 8 W: −4, 52 W: −4).

### 3.5. Safety

One serious AE (post-injection pain) and one severe AE (post-injection pain) occurred in the same patients in the PRPr group. This was considered to be possibly related to the injection procedure. No remarkable abnormalities were found in vital signs including body temperature, respiratory rate, pulse rate, and blood pressure at each time point in either group. Neurological examination showed that one patient in each group showed mild muscle weakness (more than grade 4 by manual muscle testing) in the lower extremities; however, their muscle power spontaneously recovered during the observation period.

## 4. Discussion

A clinically significant improvement in the VAS score (more than a 30% reduction [23]) was identified in both the PRPr and CS groups at eight weeks post-injection; however, there were no significant differences between the groups. This suggests that PRPr has a similar short-term analgesic effect to that of corticosteroids. Tuakli-Wosornu et al. [17] reported the effect of PRP injection for discogenic LBP patients in a double-blinded RCT and showed that the mean change in the numerical rating scale worst pain at week 8 was −2.07 (from 7.89 to 5.82). Levi et al. [33] reported that the mean change in VAS from baseline was −23.9 mm (from 64.5 to 41.5) at eight weeks after intradiscal injection of PRP in a prospective clinical trial. The change in the VAS score (−30.9 mm) in the PRPr group in our study was comparable to the change reported in the previous two clinical trials.

Autologous PRPr was isolated from all 16 patients followed by randomization; therefore, our clinical protocol was set to voluntarily receive PRPr (as an optional injection) according to the patient’s wishes after evaluating the primary outcome. As a result, 15 patients who expressed a desire for further therapeutic effects received the optional injection.

One of the CS patients significantly improved in terms of LBP and QOL at week 8 and did not wish to receive the second injection; the patient’s LBP and QOL remained satisfactory for 52 weeks. However, the long-term effects of corticosteroids in patients with discogenic pain remains controversial [34,35,36], despite one patient in our study showing a favorable therapeutic effect with the CS injection.

Therefore, evaluation of secondary outcomes after the optional injection was conducted in 15 patients. There were no significant differences in the changes and % changes in VAS and ODI in the PRPr group and the CS group over the period. The change in the RDQ score after the optional injection showed a similar tendency, and the % change in the PRPr group was more significant than that of the CS group at 24 weeks post-optional injection. The ‘walking ability’ domain of JOABPEQ showed a significant improvement in the PRPr group compared to the CS group at 4 and 8 weeks post-optional injection. Among the 15 patients who received the optional injection of PRPr, the patients who received two doses of PRPr (PRPr group) showed excellent clinical results, including more than a 70% improvement in LBP, disability, and QOL scores, and a success rate of 87.5% at the final follow-up. However, whether the double injection of PRPr has more pronounced clinical efficacy than a single injection should be evaluated in future studies with a larger number of patients.

Radiographic evaluation showed that DHI did not show a time-dependent change in either group. Similarly, MRI evaluation showed no changes in the disc degeneration grade from baseline in either group. Similar to our preliminary study [18], the radiographic findings did not indicate progression or regeneration of discs. On the other hand, intradiscal PRPr therapy did not cause any clinically important AEs, except for pain associated with the injection, which indicates that PRPr therapy is relatively safe.

There are several limitations to our study. First, the study did not include a placebo control, which could cause false-positive or false-negative results due to the effects of corticosteroids. Therefore, a placebo-controlled study is needed to precisely evaluate the outcome attributed to PRPr. Second, a single injection patient in the CS group was not included in the assessment of secondary outcomes; therefore, there is a possibility that the mean data of the CS group might be underestimated. Third, our study involved the use of PRPr [18] but not PRP itself. Therefore, it is possible that the efficacy might differ between PRPr and other variations of PRPs [16].

## 5. Conclusions

The intradiscal injection of PRPr showed clinically significant improvements in LBP intensity in patients with discogenic LBP, similar to those injected with glucocorticoid at eight weeks post-injection. PRPr treatment was safe and maintained improvements in pain, disability, and QOL during 60 weeks of follow-up.

## Figures and Tables

**Table 1:** Inclusion and exclusion criteria.

Inclusion Criteria
Patients aged more than 18 years old were included if they had:
1. Low back pain for at least 3 months.
2. Low back pain visual analogue scare (VAS) score more than 40 mm.
3. ODI score (%) more than 20% at bassline.
4. Painful degenerative disc disease at least one lumbar level from L3/L4 to L5/S1 confirmed by radiographic findings and provocative discography.
(A) Disc degeneration evaluated by MRI (more than grade II by Pfirrmann grading [19]).
(B) Less than a 50% decrease of disc height measurement by lumbar radiograph.
(C) Discogenic pain evaluated by provocative discography.
5. Provided written informed consent.
Exclusion Criteria
Patients were excluded if they had:
1. Remarkable neurological symptoms including cauda equine and neuropathy in the lower extremities.
2. Any systematic or spinal infections.
3. Undergone any lumbar surgeries.
4. Undergone any interventional intervertebral disc therapies.
5. Intervertebral instability evaluated by lumbar radiograph.
6. Spondylolisthesis (more than grade I by Meyerding classification [20]).
7. A history of neuro-muscular diseases, cerebral diseases, malignant tumor, and blood coagulation disorders.
8. Any diseases that were high risk for infections after the injection treatment.
9. Anti-coagulant or anti-platelet drugs at the time of treatment.
10. Reported that they were pregnant or lactating.
11. Difficulty in participating over the evaluation period.
12. More than 10-points in doctor version of brief scale for evaluation of psychiatric problems in orthopedic patients (BS-POP) and more than 15-points in patient version of BS-POS [21].
13. Any contraindication for MRI examination.
14. Been judged as inappropriate for clinical study by the principal investigator or co-investigators.

**Table 2:** Patients’ baseline characteristics.

	PRPr (*n* = 9)	CS (*n* = 7)	*p*-Value
Age	35.1 (8.7)	27.9 (5.2)	0.09
Gender (male: *n*, %)	6 (66.7%)	5 (71.4%)	0.78
Target disc level (*n*, %)			0.16
L3/L4	1 (9.0%)	3 (30%)	
L4/L5	5 (45.5%)	6 (60%)	
L5/S1	5 (45.5%)	1 (10%)	
Number of the target disc			0.78
One disc	7	4	
Two discs	2	3	
VAS	68.3 ± 13.3	59.4 ± 12.4	0.19
ODI (%)	36.0 ± 11.8	33.3 ± 11.6	0.66
RDQ	8.6 ± 4.8	9.3 ± 4.7	0.77
JOABPEQ			
Low back pain	22.0 ± 21.5	12.1 ± 9.9	0.28
Lumbar function	51.8 ± 23.7	59.6 ± 28.6	0.56
Walking ability	65.0 ± 28.1	66.3 ± 34.5	0.94
Social function	48.6 ± 15.2	43.7 ± 16.0	0.55
Mental health	42.2 ± 21.8	47.6 ± 17.6	0.61
Pfirrmann classification			N.A.
Grade 4	11	10	
Modified Pfirrmann classification			N.A.
Grade 4	8	5	
Grade 5	0	3	
Grade 6	3	2	
Blood cell count of whole blood			
Platelet (×10^3^/µL)	262.2 ± 45.7	250.3 ± 37.1	0.57
WBC (×10^3^/µL)	7.1 ± 1.8	5.7 ± 0.7	0.08
Blood cell count of PRP			
Platelet (×10^3^/µL)	1054.1 ± 350.3	1148.0 ± 399.8	0.63
WBC (×10^3^/µL)	0.1 ± 0.1	0.1 ± 0.2	0.99

**Table 3:** Change and % change of VAS, ODI, and RDQ from baseline.

	Change			% Change		
Week	PRPr	CS	*p*-Value	PRPr	CS	*p*-Value
VAS						
4	−19.0 ± 21.3	−34.9 ± 20.1	0.15	−29.4 ± 37.0	−57.6 ± 31.2	0.13
8	−30.9 ± 22.7	−26.3 ± 29.8	0.73	−48.2 ± 34.9	−41.7 ± 54.5	0.78
12 (4)	−38.3 ± 19.6	−32.8 ± 13.4	0.56	−60.1 ± 31.9	−56.4 ± 22.0	0.81
16 (8)	−47.9 ± 21.2	−33.3 ± 13.4	0.18	−74.2 ± 33.5	−57.3 ± 22.5	0.30
20 (12)	−45.4 ± 26.3	−29.8 ± 12.8	0.64	−67.5 ± 37.3	−68.7 ± 20.3	0.94
32 (26)	−56.8 ± 20.2	−36.8 ± 17.1	0.10	−84.2 ± 23.8	−66.6 ± 28.6	0.25
60 (52)	−53.4 ± 24.7	−36.4 ± 23.7	0.25	−78.2 ± 33.2	−61.0 ± 37.9	0.41
ODI (%)						
4	−8.2 ± 9.5	−7.2 ± 8.4	0.83	−20.4 ± 27.1	−28.5 ± 38.0	0.63
8	−14.5 ± 11.6	−7.7 ± 8.9	0.22	−37.7 ± 31.9	−31.0 ± 41.1	0.72
12 (4)	−17.9 ± 13.2	−11.2 ± 7.8	0.30	−46.5 ± 33.4	−37.1 ± 34.4	0.61
16 (8)	−23.6 ± 14.9	−11.9 ± 7.3	0.10	−62.1 ± 30.6	−39.0 ± 32.2	0.19
20 (12)	−21.9 ± 13.4	−12.7 ± 6.1	0.14	−58.9 ± 31.1	−43.4 ± 32.1	0.37
32 (26)	−26.9 ± 13.1	−14.5 ± 10.8	0.14	−74.8 ± 27.9	−45.9 ± 41.1	0.18
60 (52)	−26.6 ± 14.8	−13.9 ± 9.7	0.12	−76.0 ± 37.6	−42.4 ± 31.5	0.13
RDQ						
4	−2.2 ± 5.9	−2.3 ± 4.2	0.95	−25.6 ± 97.1	−38.0 ± 58.4	0.27
8	−3.4 ± 6.7	−1.7 ± 4.2	0.58	−39.5 ± 85.8	−32.8 ± 58.5	0.54
12 (4)	−6.9 ± 6.4	−1.6 ± 3.6	0.13	−54.8 ± 67.5	−31.0 ± 47.8	0.51
16 (8)	−6.6 ± 6.1	−1.7 ± 2.9	0.10	−56.7 ± 63.7	−25.6 ± 43.2	0.32
20 (12)	−6.7 ± 6.2	−2.3 ± 4.6	0.17	−65.7 ± 35.4	−32.8 ± 56.1	0.18
32 (26)	−8.5 ± 5.3	−3.4 ± 4.0	0.09	−88.0 ± 23.5	−42.1 ± 45.1	0.03 *
60 (52)	−8.8 ± 5.0	−4.2 ± 4.5	0.13	−92.8 ± 14.1	−49.6 ± 44.4	0.10

Change (time points − baseline) and % change ([time points − baseline]/baseline × 100) in visual analog scale (VAS), Oswestry Disability Index (ODI) [26], and Roland-Morris Disability Questionnaire (RDQ) [27,28] until 60 weeks after the injection of the platelet-rich plasma releasate (PRPr) or corticosteroid (CS). Number in parentheses indicates weeks after the optional injection. Data were expressed as means ± standard deviation (SD). * *p* < 0.05 between the groups.

## Data Availability

The data presented in this study are available on request from the corresponding author.
